# Supplementary material for: Accessible LAMP-Enabled Rapid Test (ALERT) for Detecting SARS-CoV-2
Source: Viruses. 2021 Apr 23;13(5):742. doi: 10.3390/v13050742 (PMC8146324; doi:10.3390/v13050742)
Supplement: Supplementary file 1 [file viruses-13-00742-s001.zip › viruses-1163569-supplementary 1/Viruses/Images/Table 1.pdf]

	Approximate LOD (copies/µl) for 95% TPR
RNA (Water)	25 - 50
Virus (Any)	1 - 2
Virus (1x PBS)	1 - 2
Virus (Nasal)	0.5 - 1
Virus (Saliva)	0.1 - 0.5
