# Supplementary material for: Accessible LAMP-Enabled Rapid Test (ALERT) for Detecting SARS-CoV-2
Source: Viruses. 2021 Apr 23;13(5):742. doi: 10.3390/v13050742 (PMC8146324; doi:10.3390/v13050742)
Supplement: Supplementary file 1 [file viruses-13-00742-s001.zip › viruses-1163569-supplementary 1/Viruses/Images/Table 2.pdf]

	Positive Result	n	True Negative Rate
Mock Test Samples	0	6	100.0%
Cross-Reactivity Test Samples	1	30	96.7%
Mock + Cross-Reactivity Test Samples	1	36	97.2%
