# Supplementary material for: Accessible LAMP-Enabled Rapid Test (ALERT) for Detecting SARS-CoV-2
Source: Viruses. 2021 Apr 23;13(5):742. doi: 10.3390/v13050742 (PMC8146324; doi:10.3390/v13050742)
Supplement: Supplementary file 1 [file viruses-13-00742-s001.zip › viruses-1163569-supplementary 1/Viruses/suplemental 1.pdf]

a. b. c.

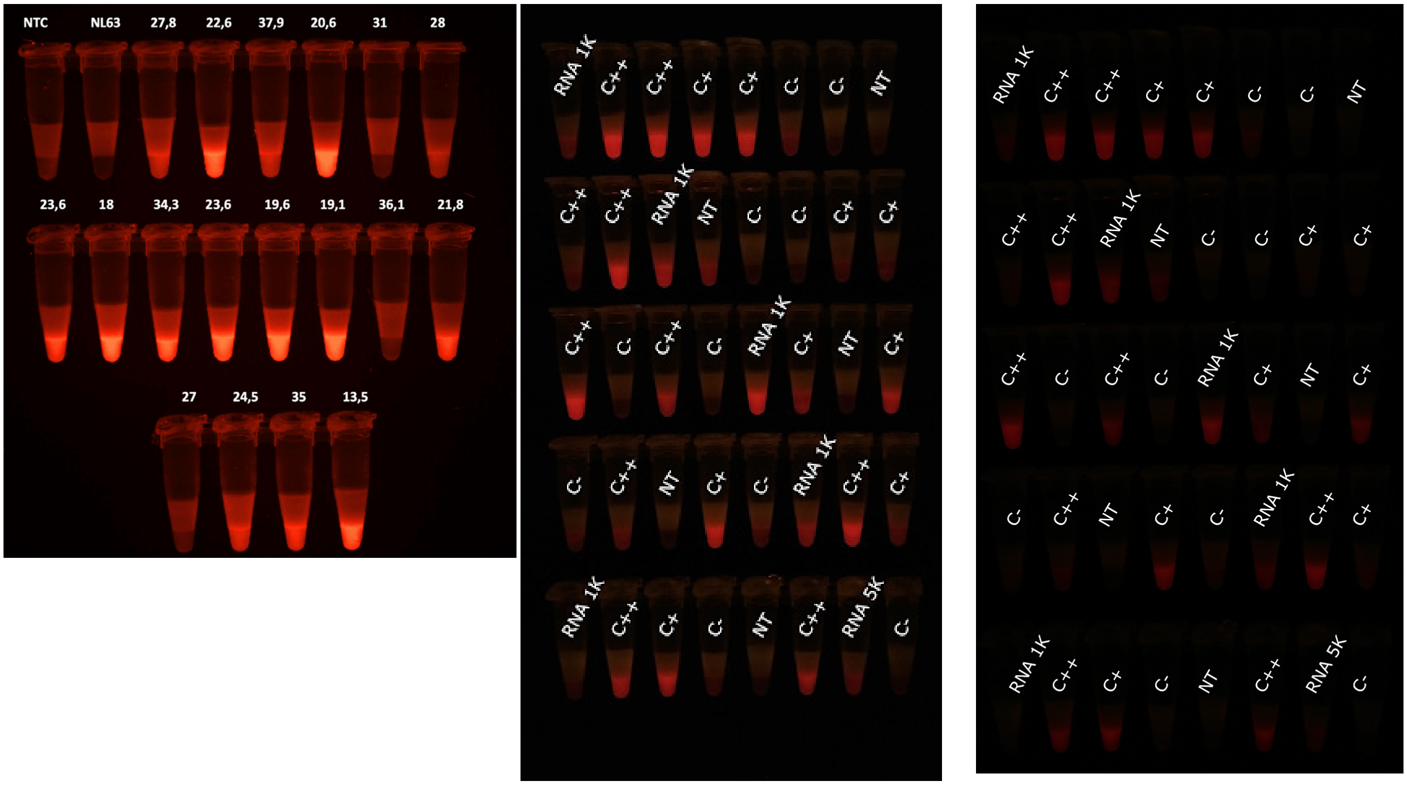

A. Validation study conducted at Hôpital Saint-Louis, Ct values are represented above detection cartridges, NL63 is cross-reactivity experiment with coronavirus NL63. **B** High exposure image of validation study conducted at Pontificia Universidad Católica de Chile, C++ represents samples with Ct below 30, C+ above 30 and C- above 40 or no amplification. RNA 1K and 5K represents positive control from in vitro transcription at 1000 or 5000 copies per reaction respectively **C.** Same tubes as B with a lower exposure image.
