# Supplementary material for: Accessible LAMP-Enabled Rapid Test (ALERT) for Detecting SARS-CoV-2
Source: Viruses. 2021 Apr 23;13(5):742. doi: 10.3390/v13050742 (PMC8146324; doi:10.3390/v13050742)
Supplement: Supplementary file 1 [file viruses-13-00742-s001.zip › viruses-1163569-supplementary 1/Viruses/Supplemental Table 2.pdf]

Sample #	Known Value	Sample Type	Sample Source	Ct Value	Result	Result Interpretation	Notes
**Validation Trial Conducted In France**							
1	Negative	Cross Reactivity	Coronavirus NL63	0	No Fluorescence	Negative	
2	Positive	Clinical	NP	27.8	Fluorescence	Positive	
3	Positive	Clinical	NP	22.6	Fluorescence	Positive	
4	Positive	Clinical	NP	37.9	Fluorescence	Positive	
5	Positive	Clinical	NP	20.6	Fluorescence	Positive	
6	Positive	Clinical	NP	31	No Fluorescence	Negative	False Negative
7	Positive	Clinical	NP	28	Fluorescence	Positive	
8	Positive	Clinical	NP	23.6	Fluorescence	Positive	
9	Positive	Clinical	NP	18	Fluorescence	Positive	
10	Positive	Clinical	NP	34.3	Fluorescence	Positive	
11	Positive	Clinical	NP	23.6	Fluorescence	Positive	
12	Positive	Clinical	NP	19.6	Fluorescence	Positive	
13	Positive	Clinical	NP	19.1	Fluorescence	Positive	
14	Positive	Clinical	NP	36.1	No Fluorescence	Negative	False Negative
15	Positive	Clinical	NP	21.8	Fluorescence	Positive	
16	Positive	Clinical	NP	27	No Fluorescence	Negative	False Negative
17	Positive	Clinical	NP	24.5	Fluorescence	Positive	
18	Positive	Clinical	NP	35	Fluorescence	Positive	
19	Positive	Clinical	NP	13	Fluorescence	Positive	
**Validation Trial Conducted In Chile**							
1	Negative	Clinical	NP	0	Faint Fluorescence	Possible positive - Retest	
2	Negative	Clinical	NP	0	No Fluorescence	Negative	
3	Negative	Clinical	NP	0	No Fluorescence	Negative	
4	Negative	Clinical	NP	0	No Fluorescence	Negative	
5	Negative	Clinical	NP	0	No Fluorescence	Negative	
6	Negative	Clinical	NP	0	No Fluorescence	Negative	
7	Negative	Clinical	NP	0	Faint Fluorescence	Possible positive - Retest	
8	Negative	Clinical	NP	0	Faint Fluorescence	Possible positive - Retest	
9	Negative	Clinical	NP	0	Faint Fluorescence	Possible positive - Retest	
10	Negative	Clinical	NP	0	No Fluorescence	Negative	
11	Positive	Clinical	NP	31.68	Fluorescence	Positive	
12	Positive	Clinical	NP	34.57	Fluorescence	Positive	
13	Positive	Clinical	NP	35.34	Faint Fluorescence	Possible positive - Retest	
14	Positive	Clinical	NP	33.4	Faint Fluorescence	Possible positive - Retest	
15	Positive	Clinical	NP	34.32	Fluorescence	Positive	
16	Positive	Clinical	NP	32.2	Fluorescence	Positive	
17	Positive	Clinical	NP	32.34	Fluorescence	Positive	
18	Positive	Clinical	NP	34.17	Faint Fluorescence	Possible positive - Retest	
19	Positive	Clinical	NP	32.55	Fluorescence	Positive	
20	Positive	Clinical	NP	20.25	Fluorescence	Positive	
21	Positive	Clinical	NP	21.22	Fluorescence	Positive	
22	Positive	Clinical	NP	26.88	Faint Fluorescence	Possible positive - Retest	
23	Positive	Clinical	NP	26.11	Fluorescence	Positive	
24	Positive	Clinical	NP	24.16	Fluorescence	Positive	
25	Positive	Clinical	NP	27.68	Fluorescence	Positive	
26	Positive	Clinical	NP	29.29	Faint Fluorescence	Possible positive - Retest	
27	Positive	Clinical	NP	23.63	Fluorescence	Positive	
28	Positive	Clinical	NP	20.85	Fluorescence	Positive	
29	Positive	Clinical	NP	28.1	Fluorescence	Positive	

Data from clinical validation conducted in France and Chile
