# Supplementary material for: Accessible LAMP-Enabled Rapid Test (ALERT) for Detecting SARS-CoV-2
Source: Viruses. 2021 Apr 23;13(5):742. doi: 10.3390/v13050742 (PMC8146324; doi:10.3390/v13050742)
Supplement: Supplementary file 1 [file viruses-13-00742-s001.zip › viruses-1163569-supplementary 1/Viruses/Supplementl Table 1.pdf]

Supplemental table 1: Primers

SARS-CoV-2 and Influenza B primers were found from the literature but modified for the sequence specific QUASR reporting system by us according to Ball et al. 2016. A gene with nasopharyngeal specific expression (Saferali et al. 2020), BPI fold containing family A, member 1 (BPIFA1), was used to design a sampling and process control using Primer Explorer v.4 (Eiken Chemical Co., Japan) to traverse exon/exon boundaries and therefore be mRNA specific (FIGURE). Primers were synthesized by IDT (Coraville, Iowa) and LGC Biosearch (Petaluma, CA). Primers modified with quenchers and TexasRed were purified by HPLC while all other primers were prepared by desalting. Lyophilized primers were reconstituted in water.

**BPIFA1***	
FIP	ACAGCAGGCCATTGCTGAGGCTGCCCTTGAGTCCCACA
BIP	GAAAACCTTCCGCTCCTGGACATCCAAGCAGTCCCCCAAG
LF	GTCAAGCTTCCTGCAAGACC
LB-F	FAM-TCCTGAAGCCTGGAGGAGG
LB-Q	GGCTTCAGGA-BHQ1
F3	CCCTGCCCTTGAATGTGAAT
B3	GGCCAGGAATCACTGACG
SARS-CoV-2 **NB/NA**	*Zhang et al. 2020*
FIP	TCTGGCCCAGTTCCTAGGTAGTTCGTGGTGGTGACGGTAA
BIP	AGACGGCATCATATGGGTTGCACGGGTGCCAATGTGATCT
LB	ACTGAGGGAGCCTTGAATACA
LF-TX**	5TexRd-CCATCTTGGACTGAGATCTTTCATT
LF-Q	CAGTCCAAGATGG-3IAbRQSp
F3	ACCGAAGAGCTACCAGACG
B3	TGCAGCATTGTTAGCAGGAT
NA-F3	TGGCTACTACCGAAGAGCT
**Influenza B**	*Ge et al. 2017*
FIP	TMARGGACAATACATTACGCATATCGATAAAGGAGGAAGTAAACACTCA
BIP	TAAAYGGAACATTCCTCAAACACCACTCTGGTCATAGGCATTC
LF	TYAAACGGAACTTCCCTTCTTTC
LB-F	FAM-GGATACAAGTCCTTATYAACTCTGC
LB-Q	AAGGACTTGTATTCC-BHQ1
F3	AGGGACATGAACAACAAAGA
B3	CAAGTTTAGCAACAAGCCT

SARS-CoV-2 **NM**	*Broughton et al. 2020*
FIP-F	FAM-TGCGGCCAATGTTTGTAATCAGCCAAGGAAATTTTGGGGAC
FIP-Q	CATTGGCCGCA-IABkFQ
BIP	CGCATTGGCATGGAAGTCACTTTGATGGCACCTGTGTAG
LF	TTCCTTGTCTGATTAGTTC
LB	ACCTTCGGGAACGTGGTT
F3	AACACAAGCTTTCGGCAG
B3	GAAATTTGGATCTTTGTCATCC

All sequences presented 5’ to 3’
Y represents degenerate C or T; R represents degenerate A or G; M represents degenerate A or C
FAM=Fluorescein
TxRed=TexasRed
BHQ1= Black Hole Quencher 1
IAbRQSp= Iowa Black RQ-Sp
IABkFQ= Iowa Black FQ
* When used with NEB Colorimetric LAMP kit, a non-modified version of LB-F was used and LB-Q was omitted.
**In one iteration of this primer a FAM fluorophore is used at the 5’ end.
