# Supplementary figures and images for: Accessible LAMP-Enabled Rapid Test (ALERT) for Detecting SARS-CoV-2
Source: Viruses. 2021 Apr 23;13(5):742. doi: 10.3390/v13050742 (PMC8146324; doi:10.3390/v13050742)

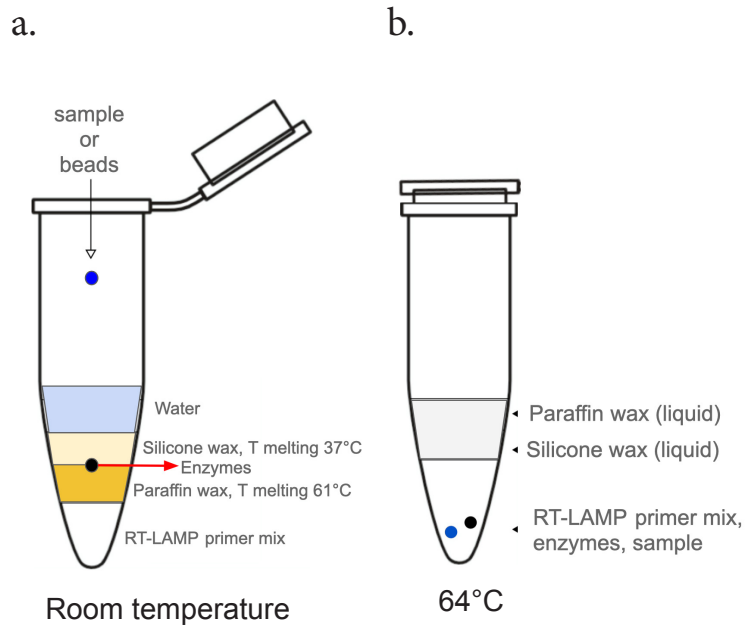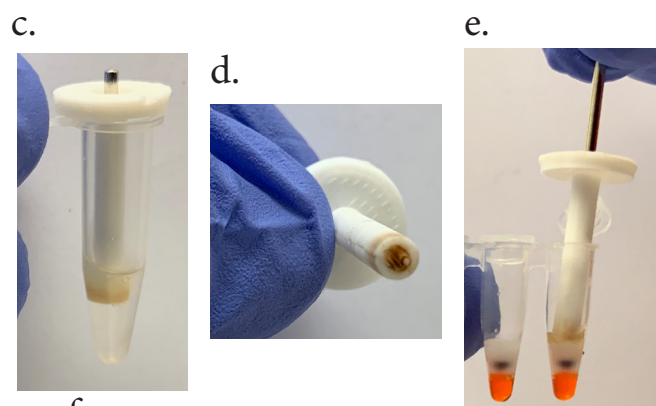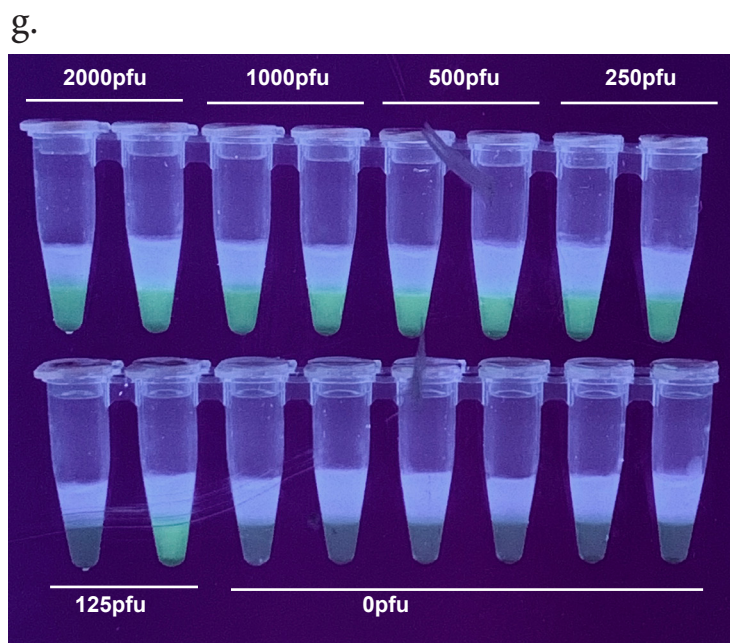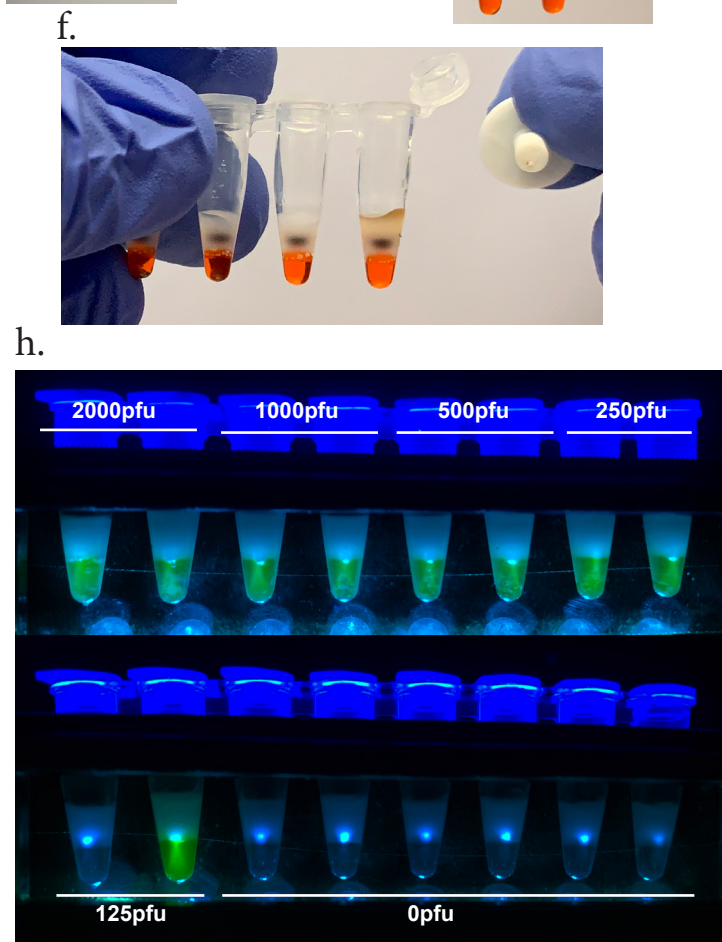

Supplement: Supplementary file 1 [file viruses-13-00742-s001.zip › viruses-1163569-supplementary 1/Viruses/Images/Figure 1.pdf]

a.

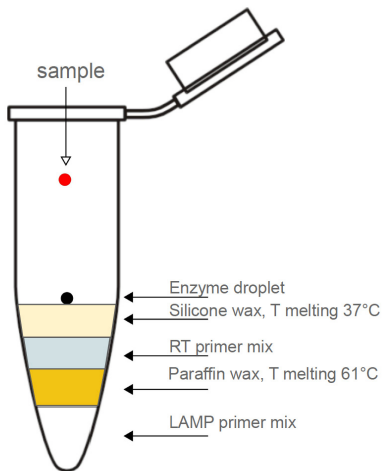

Room temperature

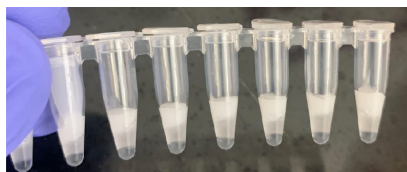

b.

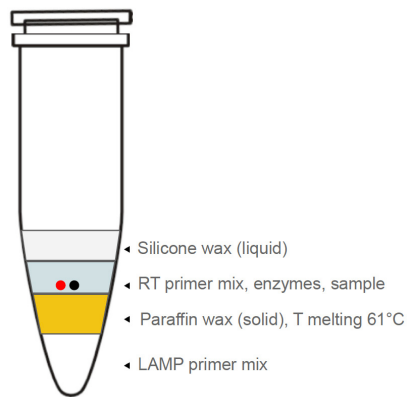

55°C

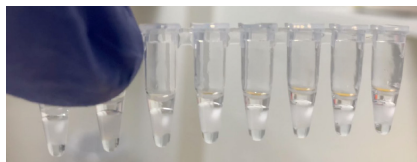

c.

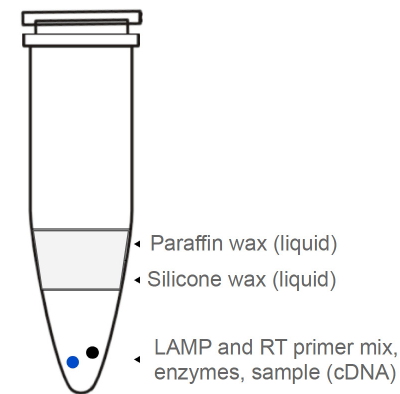

63°C

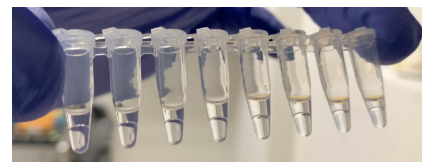

d.

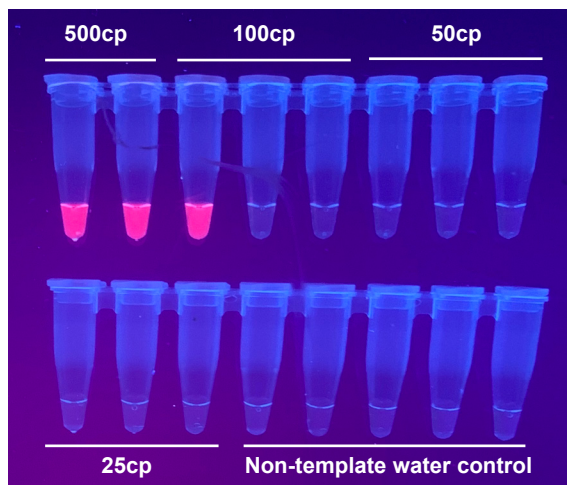

e.

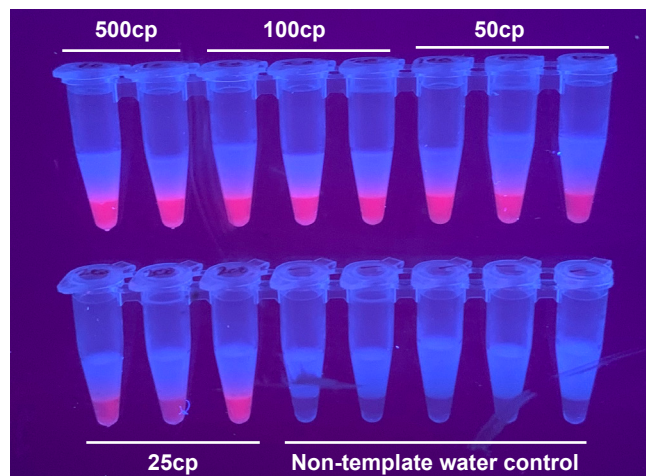

Supplement: Supplementary file 1 [file viruses-13-00742-s001.zip › viruses-1163569-supplementary 1/Viruses/Images/Figure 2.pdf]

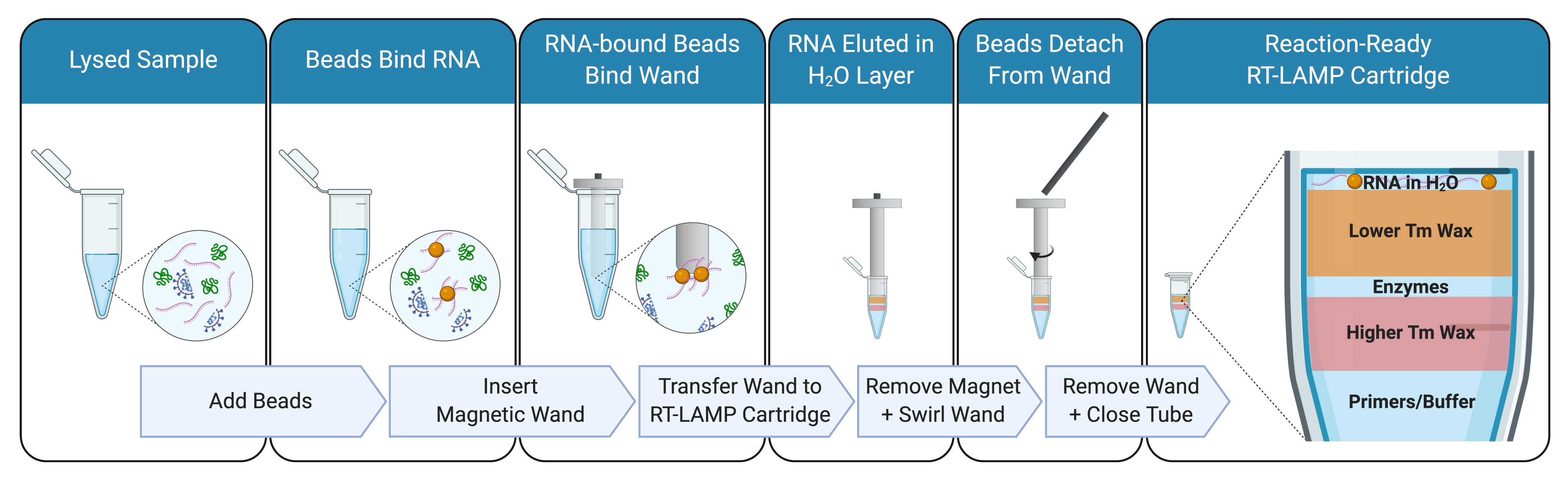

Supplement: Supplementary file 1 [file viruses-13-00742-s001.zip › viruses-1163569-supplementary 1/Viruses/Images/Figure 3(1).png]

a.

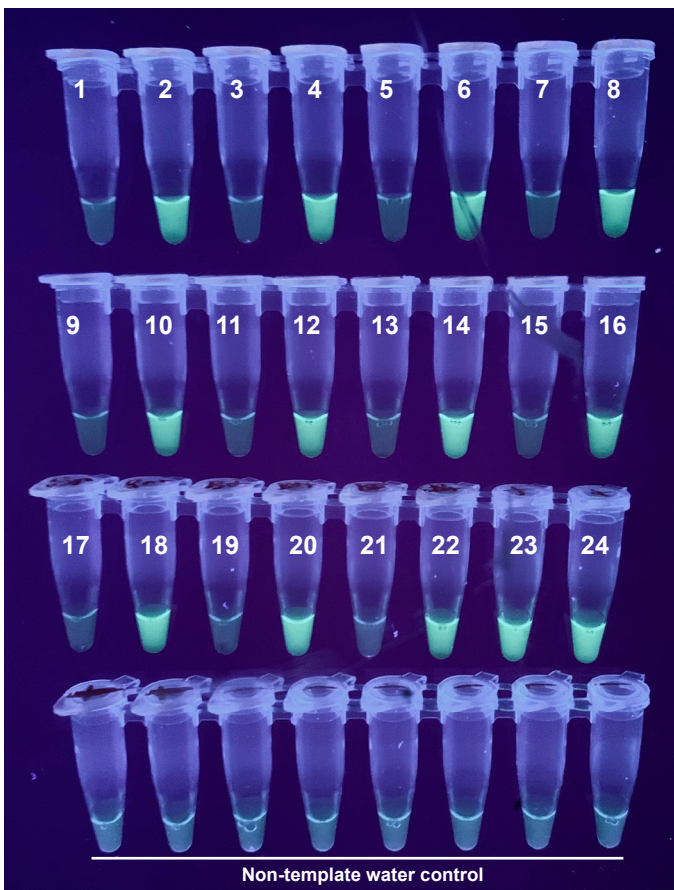

b.

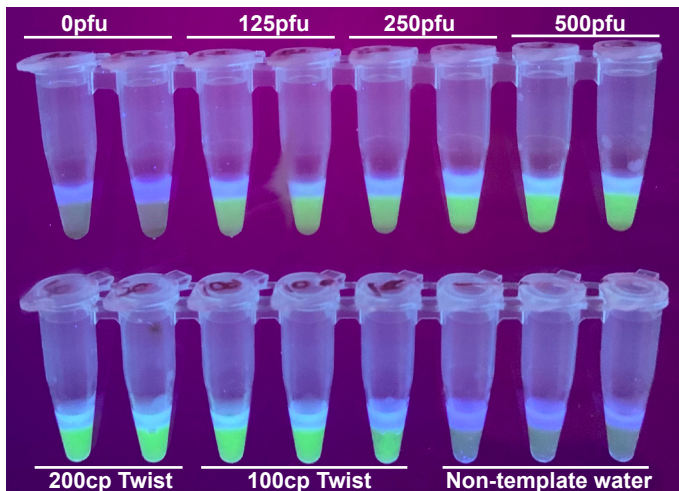

Supplement: Supplementary file 1 [file viruses-13-00742-s001.zip › viruses-1163569-supplementary 1/Viruses/Images/Figure 4.pdf]

a.

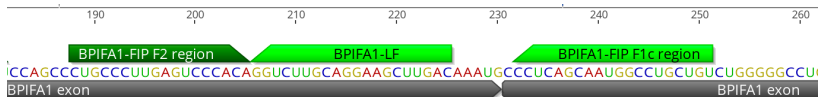

b.

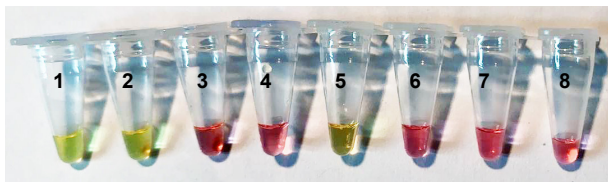

c.

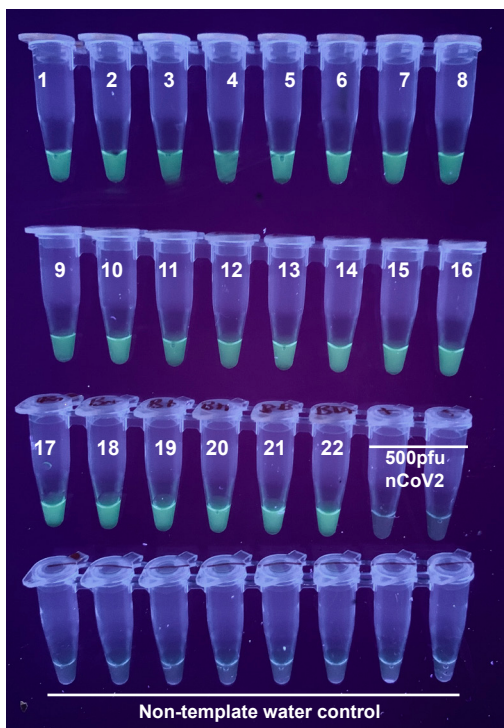

Supplement: Supplementary file 1 [file viruses-13-00742-s001.zip › viruses-1163569-supplementary 1/Viruses/Images/Figure 5.pdf]

a.

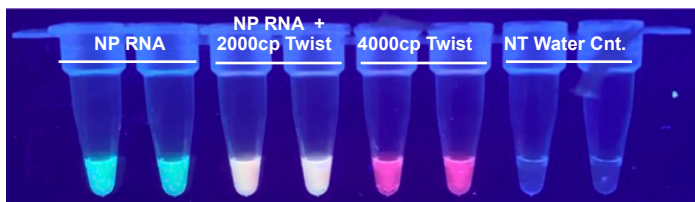

b.

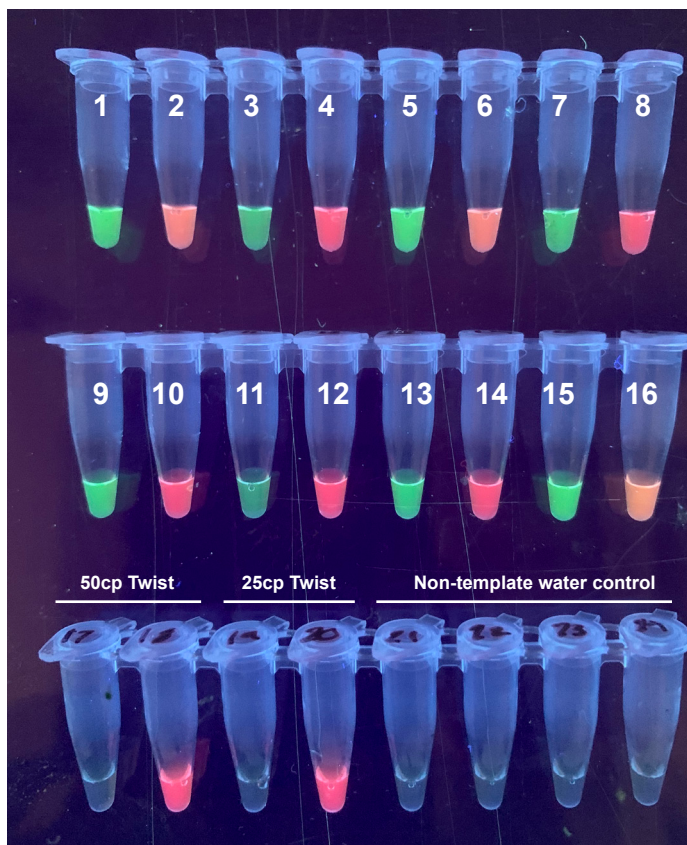

c.

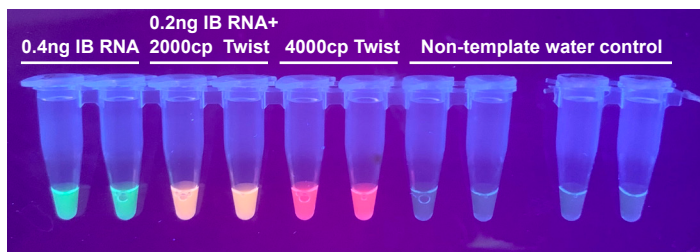

Supplement: Supplementary file 1 [file viruses-13-00742-s001.zip › viruses-1163569-supplementary 1/Viruses/Images/Figure 6.pdf]

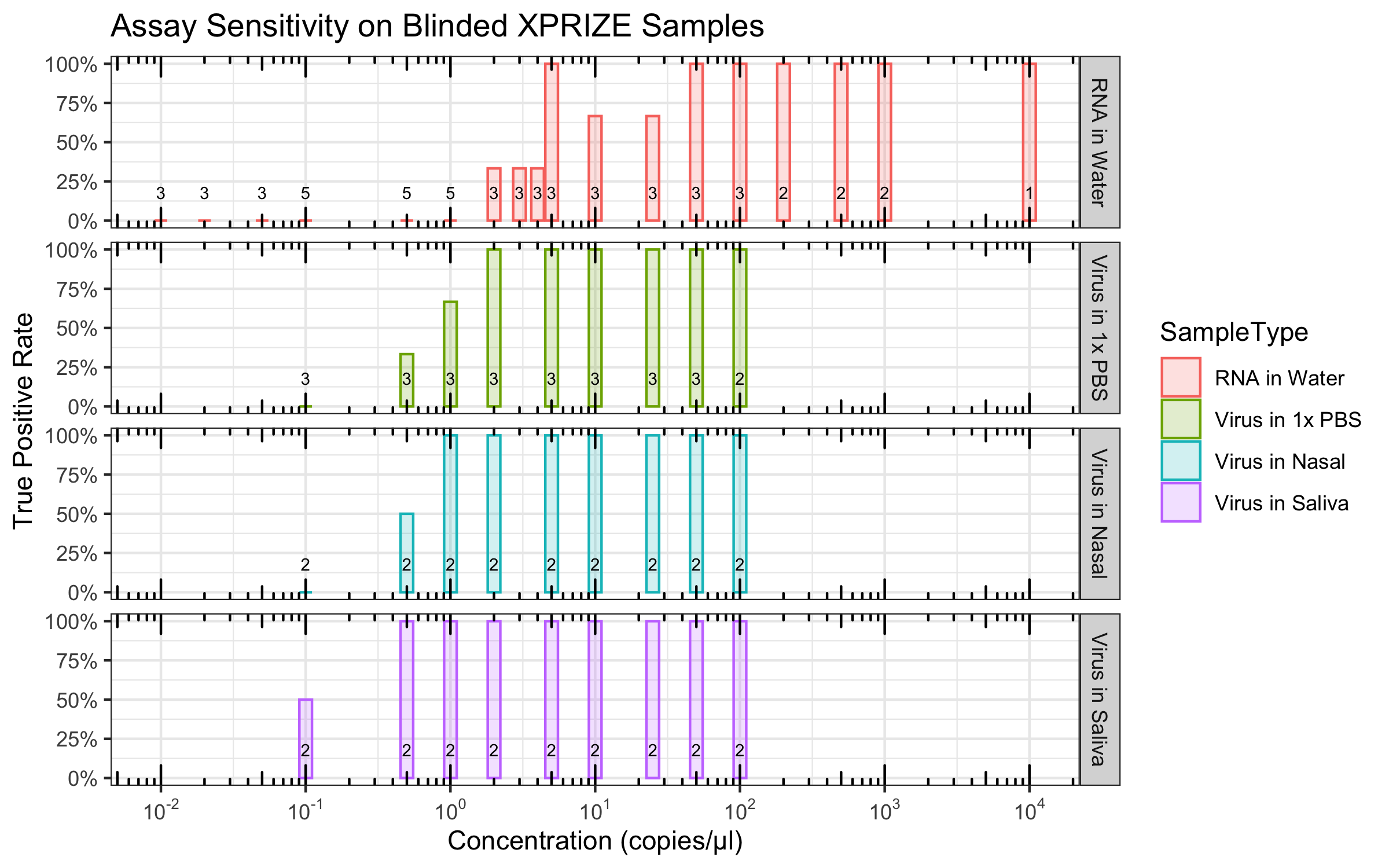

Supplement: Supplementary file 1 [file viruses-13-00742-s001.zip › viruses-1163569-supplementary 1/Viruses/Images/Figure 7.png]
