# Supplementary material for: Accessible LAMP-Enabled Rapid Test (ALERT) for Detecting SARS-CoV-2
Source: Viruses. 2021 Apr 23;13(5):742. doi: 10.3390/v13050742 (PMC8146324; doi:10.3390/v13050742)
Supplement: Supplementary file 1 [file suplemental figure 2.pdf › viruses-1163569-supplementary 1/Viruses/suplemental figure 2.pdf]

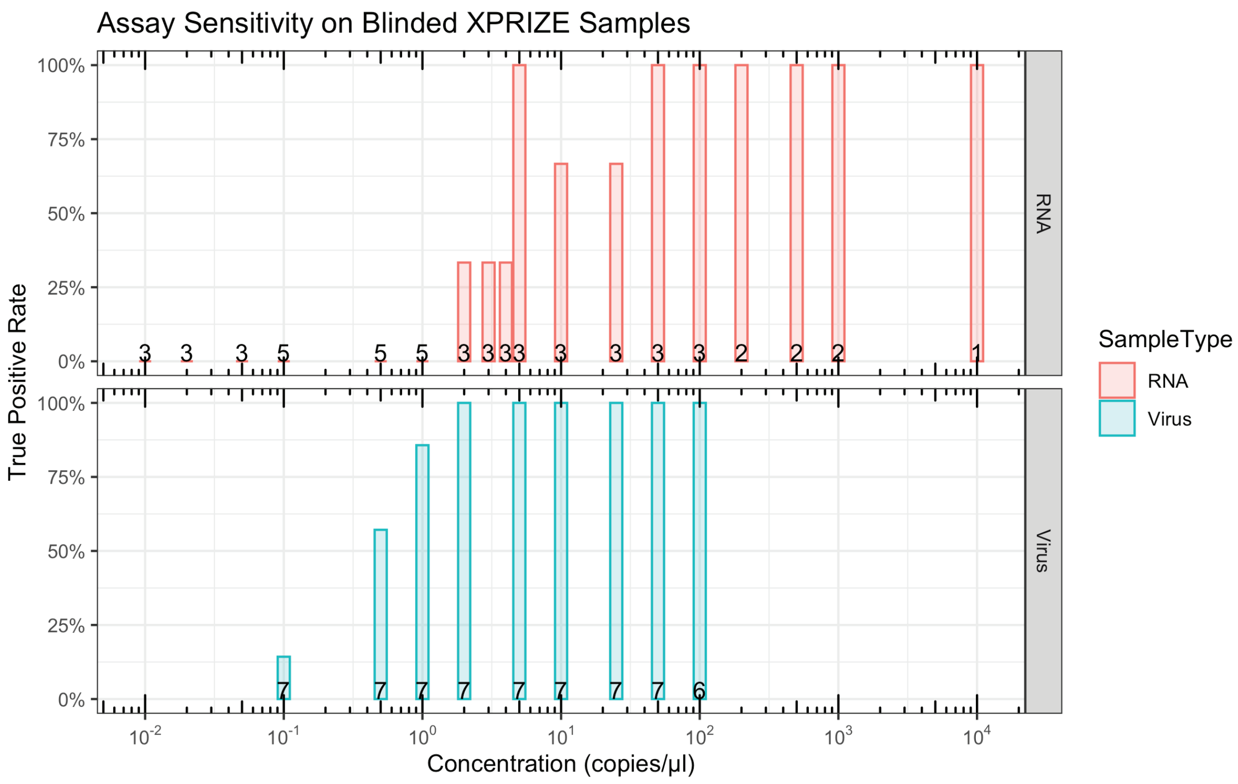

Assay sensitivity on blinded XPRIZE samples for RNA vs virus
True positive rate for our assay (y-axis) is shown for different concentrations (x-axis) for RNA vs virus. The number of samples tested at each concentration for a sample type is represented by the values above the x-axes.
